# Systemic Sclerosis Patients Present Alterations in the Expression of Molecules Involved in B-Cell Regulation

**DOI:** 10.3389/fimmu.2015.00496

**Published:** 2015-09-29

**Authors:** Lilian Soto, Ashley Ferrier, Octavio Aravena, Elianet Fonseca, Jorge Berendsen, Andrea Biere, Daniel Bueno, Verónica Ramos, Juan Carlos Aguillón, Diego Catalán

**Affiliations:** ^1^Programa Disciplinario de Inmunología, Instituto de Ciencias Biomédicas (ICBM), Facultad de Medicina, Universidad de Chile, Santiago, Chile; ^2^Hospital Clínico, Universidad de Chile, Santiago, Chile; ^3^Millennium Institute on Immunology and Immunotherapy, Santiago, Chile

**Keywords:** regulatory B cells, systemic sclerosis, IL-10, FcγRIIb, Siglec

## Abstract

The activation threshold of B cells is tightly regulated by an array of inhibitory and activator receptors in such a way that disturbances in their expression can lead to the appearance of autoimmunity. The aim of this study was to evaluate the expression of activating and inhibitory molecules involved in the modulation of B cell functions in transitional, naive, and memory B-cell subpopulations from systemic sclerosis patients. To achieve this, blood samples were drawn from 31 systemic sclerosis patients and 53 healthy individuals. Surface expression of CD86, MHC II, CD19, CD21, CD40, CD22, Siglec 10, CD35, and FcγRIIB was determined by flow cytometry. IL-10 production was evaluated by intracellular flow cytometry from isolated B cells. Soluble IL-6 and IL-10 levels were measured by ELISA from supernatants of stimulated B cells. Systemic sclerosis patients exhibit an increased frequency of transitional and naive B cells related to memory B cells compared with healthy controls. Transitional and naive B cells from patients express higher levels of CD86 and FcγRIIB than healthy donors. Also, B cells from patients show high expression of CD19 and CD40, whereas memory cells from systemic sclerosis patients show reduced expression of CD35. CD19 and CD35 expression levels associate with different autoantibody profiles. IL-10^+^ B cells and secreted levels of IL-10 were markedly reduced in patients. In conclusion, systemic sclerosis patients show alterations in the expression of molecules involved in B-cell regulation. These abnormalities may be determinant in the B-cell hyperactivation observed in systemic sclerosis.

## Introduction

Systemic sclerosis (SSc) is a systemic autoimmune disease characterized by an excessive deposition of extracellular matrix on skin and internal organs, vasculopathy, and the presence of a wide spectrum of autoantibodies. This disease is classified into limited cutaneous (lcSSc) and diffuse cutaneous (dcSSc) according to the degree of skin sclerosis, the presence of interstitial lung disease or pulmonary arterial hypertension, and the autoantibody profile (1). Fibrosis in SSc is produced by a dysregulated reparation process, marked by the differentiation of tissue fibroblasts to myofibroblasts (2). When the gene expression pattern of SSc fibroblasts was compared with that of healthy individuals, no major differences were observed, which suggests that fibrosis could be caused by exogenous stimuli, such as those provided by the immune system (3). In that work, an increase in the expression of B cell-related genes together with an infiltration of CD20^+^ B cells was observed in SSc skin, suggesting a pathogenic role of B cells.

Like most cells of the immune system, B cells express a wide array of activating and inhibitory receptors that modulate their activation status, allowing protective but controlled humoral immune responses. Abnormalities in the expression or function of these receptors on B cells have been reported in murine models of autoimmunity or in patients with autoimmune diseases (4–7). CD19 is a cell-surface signal transduction molecule that forms a complex with CD21, CD81, and CD225. CD21, or complement receptor 2 (CR2), binds to cleavage products of C3 complement component and conveys signals through CD19, thereby lowering the threshold for B-cell activation. CD19 also strengthens signals generated by the B-cell antigen receptor (BCR) and by CD40, which is activated by CD40 ligand (CD40L)-expressing T cells (8–10). Another complement receptor that is expressed on B cells – CD35 (CR1) – has been proposed to deliver inhibitory signals, thus opposing CD21 signals in the regulation of B-cell activation (11).

Fcγ receptor IIB (FcγRIIB) and members of sialic acid-binding immunoglobulin-type lectins (Siglecs) are other inhibitory receptors. FcγRIIB, a low-affinity receptor for the Fc fragment of immunoglobulin G (IgG), conveys inhibitory signals when cross-linked by IgG-containing immune complexes (12). Moreover, Siglecs are a family of immune receptors that recognize sialic acids attached to proteins. B cells express only two of them, CD22 (Siglec 2) and Siglec 10. Upon activation, CD22 recruits phosphatases that dephosphorylate several proteins, such as CD19, thus switching off B-cell activation. Although less studied, Siglec 10 presumably mediates inhibitory signals in a similar way to CD22 (4).

The multidimensional role of B cells in systemic autoimmune diseases has been progressively recognized (13). Increasing evidence suggests that B cells contribute to autoimmune responses by a series of antibody-independent mechanisms, which include antigen presentation to T cells and proinflammatory cytokine secretion (14). More recently, a population of IL-10-producing B cells with the ability to suppress autoimmune responses has been characterized in humans (15). These so-called regulatory B cells, which are enriched within the subpopulation of transitional B cells – immature B cells in transition to secondary lymphoid organs, have been shown to be numerically and/or functionally disturbed in patients with systemic autoimmune diseases, such as rheumatoid arthritis (RA) and systemic lupus erythematosus (SLE) (16, 17).

The present study was aimed at evaluating whether B-cell subpopulations from SSc patients, including transitional B cells, present alterations in frequency, phenotype, and/or expression of activating and inhibitory receptors compared with those from healthy subjects.

## Materials and Methods

### Study subjects

Thirty-one patients meeting the American College of Rheumatology criteria for SSc (18) and 53 healthy controls were recruited. The involvement of different systems or organs was evaluated with the Modified Medsger scale (19). Table 1 shows the main clinical and demographic characteristics of both groups. Blood samples (50 ml) were drawn by venous puncture for B-cell phenotyping. Due to the limited amount of sample, only some parameters could be assessed for each individual. For the expression levels of CD86, CD40, major histocompatibility class II (MHC II) molecules, CD35, CD21, CD22, and Siglec 10 on B cells, sex- and age-matched SSc patients and healthy controls groups were compared. The study was approved by the Ethical Committees of the Hospital Clínico and Facultad de Medicina, Universidad de Chile, and all subjects gave written informed consent in accordance with the Declaration of Helsinki.

**Table 1:** **Main demographic and clinical characteristics of the systemic sclerosis patients and healthy controls recruited for this study**.

Characteristics	Patients (*n* **=** 31)	Controls (*n* **=** 53)
Female/male	23/8	28/25
Age	49.3 ± 11.8	40.0 ± 13.7
Disease duration, months (mean ± SD)	102.2 ± 107.2	
lcSSc/dcSSc	22/9	
Rodnan score (mean ± SD)	13.9 ± 6.0	
ANA positivity, *n* (%)	31 (100)	
ANA pattern,^a^*n* (%)		
Speckled	10 (32.3)	
Nucleolar	8 (25.8)	
Homogeneous	9 (29.0)	
Centromere	14 (45.2)	
Anti-Scl-70 positivity, *n* (%)	6 (19.4)	
Organ involvement,^b^*n* (%)		
Peripheral vascular	16 (51.6)	
Skin	29 (93.5)	
Gastrointestinal tract	27 (90.0)	
Lung	21 (70)	
Heart	16 (51.6)	
Kidney	4 (12.9)	
Therapy		
Prednisone	3/31	
Azathioprine + prednisone	2/31	
Methotrexate	3/31	
d-penicillamine	1/31	
Methotrexate + d-penicillamine	1/31	
Methotrexate + d-penicillamine + prednisone	1/31	
Hydroxychloroquine	4/31	
Methotrexate + hydroxychloroquine	1/31	
Only symptomatic treatment	15/31	

*SD, standard deviation; lcSSc, limited cutaneous systemic sclerosis; dcSSc, diffuse cutaneous systemic sclerosis; ANA, antinuclear antibodies*.

*^a^Some patients have more than one pattern*.

*^b^Defined as a Modified Medsger scale value 1*.

### B-cell phenotyping

We characterized B cells using the following monoclonal anti-human antibodies: anti-CD19 FITC, CD19 Alexa Fluor 700, CD24 PE-Cy7, CD38 APC, CD27 APC, CD86 PE-Cy5, CD25 PE-Cy7, CD1d PE, CD21 PE, CD22 PE, CD35 PE, Siglec 10 PE (Biolegend, USA), CD40 FITC, IL-10 PE, MHC II APC eFluor 780 (eBioscience, USA), and FcγRII PE (clone 7.3; Fitzgerald Industries International, USA). For the cell surface staining procedure, peripheral blood mononuclear cells (PBMCs) were obtained from blood samples by density gradient centrifugation with Lymphoprep (Stemcell Technologies, Canada) and either stained freshly or cryopreserved in liquid nitrogen until use. Cells were incubated with fluorochrome-labeled antibodies for 30 min at 4°C, washed, and fixed before acquisition on a FACSCalibur or FACSAria III flow cytometer (BD Biosciences). Data were analyzed with FloJo 7.6 Software (USA).

For cytokine production assays, untouched B cells were isolated from whole blood (EasySep, Stemcell Technologies) and cultured in RPMI 1640 medium supplemented with 10% fetal bovine serum (HyClone, Thermo Scientific, USA) at 1 × 10^6^ cells/ml in 96-well plates with 50 ng/ml phorbol 12-myristate 13-acetate (PMA), 1 μg/ml ionomycin (Sigma-Aldrich, USA), and 1 μg/ml brefeldin A (eBioscience) for 5 h at 37°C and 5% CO_2_. For intracellular detection of IL-10 production on B-cell subpopulations, cells were stained with anti-CD19, anti-CD38, and anti-CD24 antibodies, fixed and permeabilized with Permeabilization Buffer (eBioscience), and incubated with an anti-IL-10 antibody for flow cytometry analysis. Fluorescence minus one (FMO) staining controls were used to exclude non-specific background staining. Culture supernatants were collected for detection of IL-10 and IL-6 levels by ELISA (eBioscience).

### Statistical analyses

All the study variables were tested for normal distribution with the D’Agostino-Pearson omnibus test. Differences between SSc patients and healthy control groups, or between groups of patients, were analyzed using the two-tailed unpaired Student’s *t*-test or Mann–Whitney *U* test, when appropriate. For matched groups, the two-tailed paired Student’s *t*-test or Wilcoxon signed-rank test were used, when appropriate. To examine the relationship between continuous variables, linear regression analyses were performed. For statistic analyses and graphics, Stata 12 and GraphPad Prism 5 softwares were used.

## Results

### Altered frequencies of B-cell subpopulations in peripheral blood of systemic sclerosis patients

**Figure 1:**
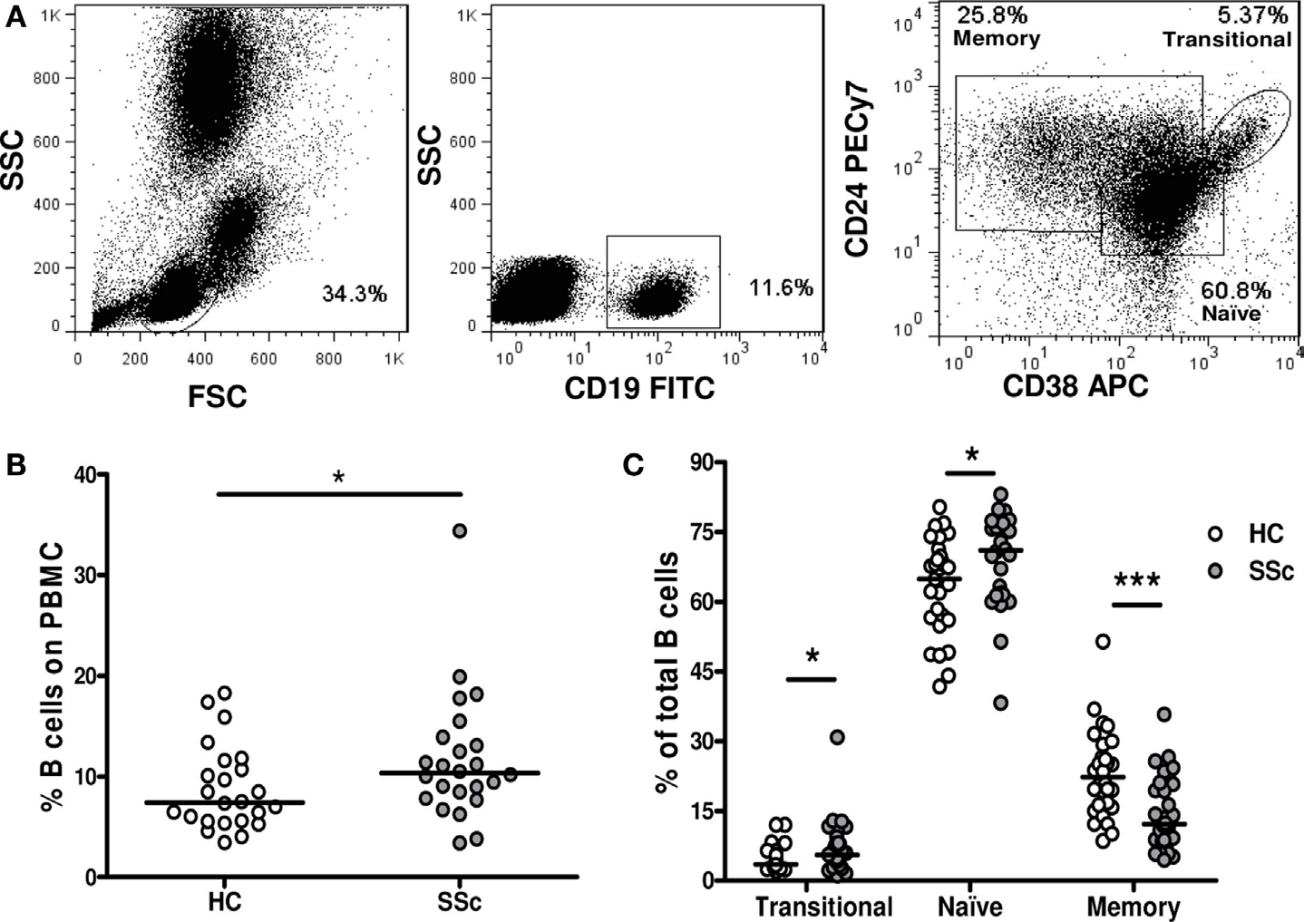
**Frequencies of B-cell subpopulations in systemic sclerosis patients**. **(A)** Flow cytometry gating strategy to identify transitional B cells (CD19^+^CD24^high^CD38^high^), naive B cells (CD19^+^CD24^int^CD38^int^), and memory B cells (CD19^+^CD24^high^CD38^−^). **(B)** Percentage of CD19^+^ B cells within peripheral blood mononuclear cells (PBMC) in healthy controls (HC) (*n* = 24) and systemic sclerosis (SSc) patients (*n* = 24). **(C)** Percentage of transitional, naive, or memory B-cell subpopulations within total CD19^+^ B cells in HC (white circles) (*n* = 31) and SSc patients (gray circles) (*n* = 30). *P < 0.05, ***P < 0.001, Mann–Whitney *U* test.

To investigate whether the frequency of different B-cell subpopulations is altered in peripheral blood of SSc patients, we analyzed them by flow cytometry. A region was set to define the lymphocytic population according to forward and side scatter patterns. B cells were defined as CD19^+^ cells, and a second region was set for them. Finally, CD24 and CD38 expression was used to discriminate transitional (CD24^high^CD38^high^) from naive (CD24^int^CD38^int^) and memory (CD24^high^CD38^−^) B cells, as previously described (16) (Figure 1A).

An increased percentage of CD19^+^ B cells was found in PBMC of SSc patients compared with healthy controls (Figure 1B). Since the relative frequency of memory B cells was dramatically decreased within SSc patients’ B cells, the observed increase in the percentage of total B cells can be explained by an expansion of naive B cells. Interestingly, the percentage of transitional B cells among total B cells was also increased in the peripheral blood of SSc patients compared with healthy subjects (Figure 1C).

### B cells from systemic sclerosis patients exhibit an activated phenotype

**Figure 2:**
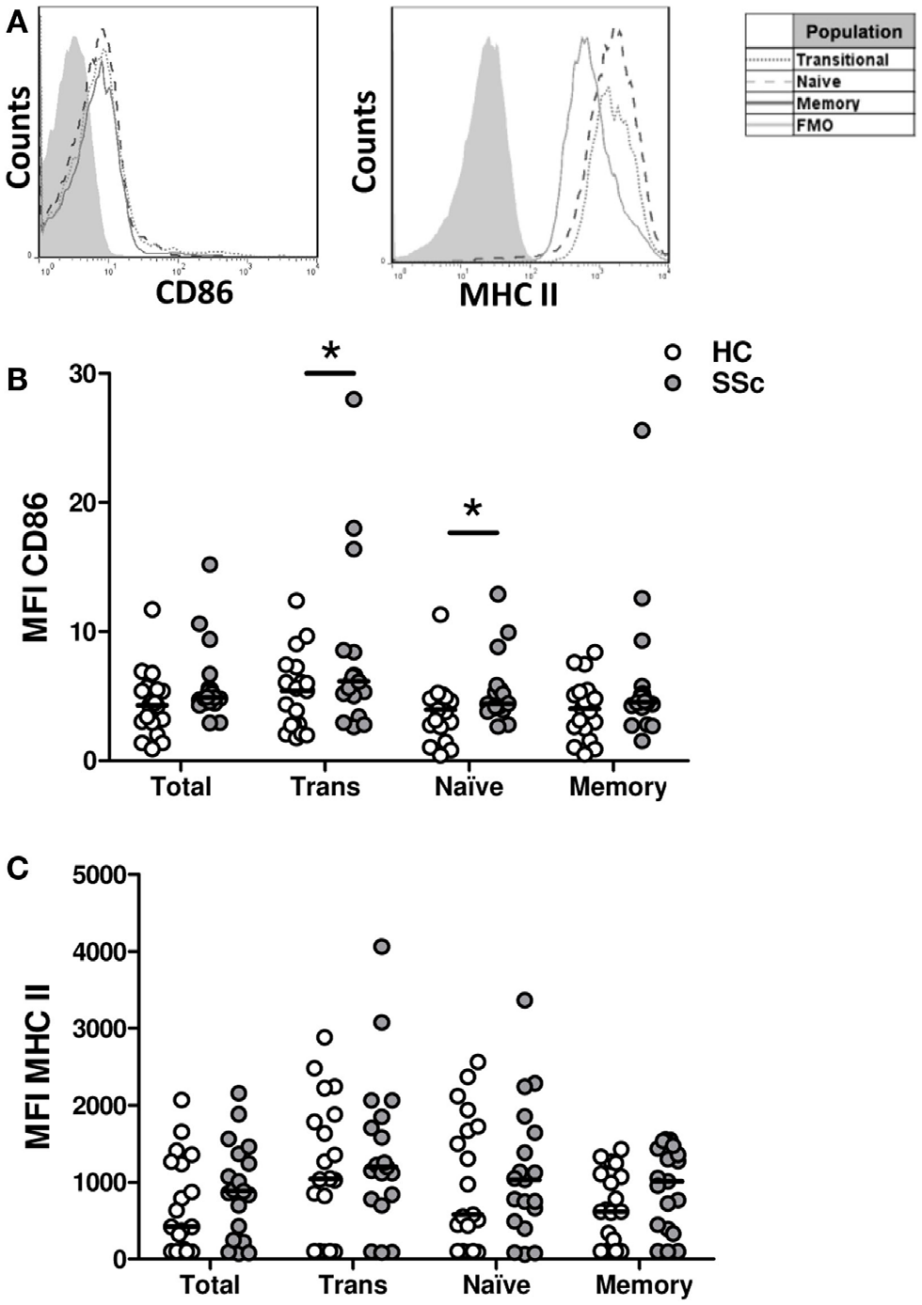
**Surface expression of CD86 and major histocompatibility class II (MHC II) molecules on B cells from systemic sclerosis patients**. **(A)** Representative histograms of the expression of CD86 and MHC II on transitional (dotted line), naive (dashed line), or memory B cells (solid line). The shaded curve represents the fluorescence minus one (FMO) control staining. **(B,C)** Expression of CD86 **(B)** and MHC II **(C)** on total CD19^+^ B cells, transitional B cells (Trans), naive B cells and memory B cells in healthy controls (HC, white circles) (*n* = 19) and systemic sclerosis patients (SSc, gray circles) (*n* = 19). *P < 0.05, Wilcoxon signed-rank test. MFI, mean fluorescence intensity.

To evaluate whether B cells from SSc patients exhibit an activated phenotype, the surface expression of MHC II and CD86 molecules, involved in antigen presentation and costimulation, respectively, and upregulated upon B-cell activation, was measured (Figure 2). Although very low, the expression of CD86 was elevated in B cells from SSc patients, particularly in the transitional and naive B-cell subpopulations, when compared with healthy subjects (Figure 2B). In contrast, no differences were observed in MHC II expression (Figure 2C).

**Figure 3:**
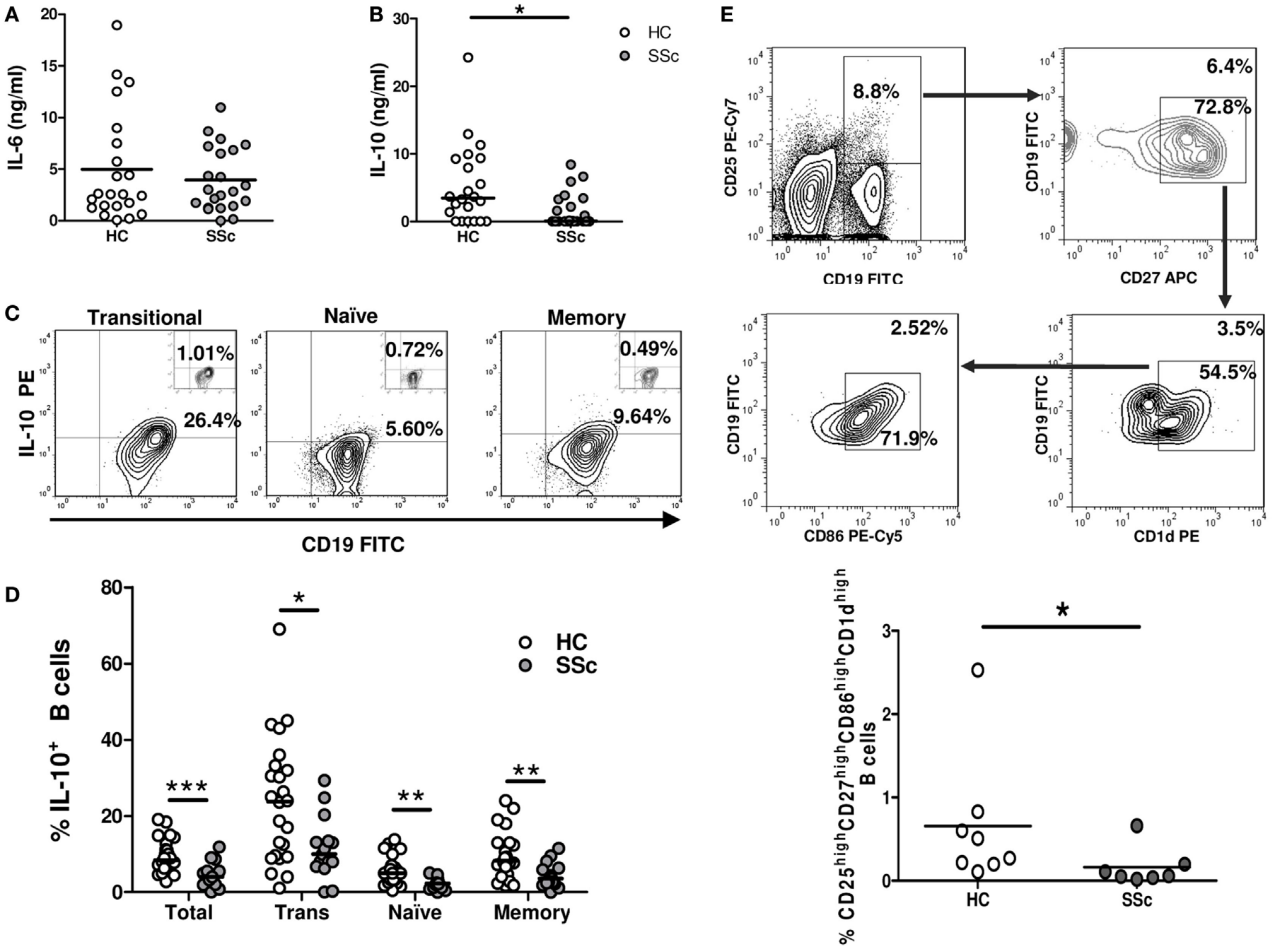
**Reduced IL-10-expressing B-cell frequencies in systemic sclerosis patients**. **(A–D)** Isolated B cells from healthy controls (HC) or systemic sclerosis patients (SSc) were stimulated for 5 h with PMA and ionomycin. IL-6 levels **(A)** and IL-10 levels **(B)** secreted by B cells from HC (*n* = 22) or SSc (*n* = 22) were determined by ELISA and compared with the Mann–Whitney *U* test. **(C)** Representative plots of the percentage of CD19^+^IL-10^+^ B cells within transitional (left), naive (middle), and memory (right) populations. The small inserts on each plot represent the background percentages, as determined by the fluorescence minus one (FMO) control staining. **(D)** Graph summarizing the percentages of CD19^+^IL-10^+^ cells among total, transitional (Trans), naive, and memory B cells in HC (white circles) (*n* = 24) and SSc (gray circles) (*n* = 15) groups. Statistic comparisons were made using the unpaired Student’s *t*-test. **(E)** Peripheral blood mononuclear cells from HC (*n* = 8) and SSc patients (*n* = 7) were stained and analyzed for the presence of CD25^high^CD27^high^CD86^high^CD1d^high^ B cells and compared with the Mann–Whitney *U* test. The upper panel represents the gating strategy to identify the subpopulation. Numbers outside and inside the gates indicate the percentages of gated cells from the total or previously gated B cells, respectively. *P < 0.05, **P < 0.01, ***P < 0.001.

IL-6 and IL-10 are two B-cell-secreted cytokines that have been involved in the SSc fibrotic process (2). These cytokines were assessed in isolated and stimulated B cells as an estimation of their activation status. No differences were observed in the levels of IL-6 secreted by B cells from SSc patients and healthy controls (Figure 3A). However, SSc patients exhibited a significantly lower IL-10 secretion in comparison to healthy subjects (Figure 3B). To explore which B-cell subpopulation was responsible for this decrease in IL-10 production, intracellular IL-10 expression was evaluated by flow cytometry. The majority of IL-10^+^ cells was found within transitional B cells, both in SSc patients and healthy controls, which is in accordance with previous reports (Figure 3C) (16). Of note, the percentage of IL-10^+^ B cells was reduced in all B-cell subpopulations of SSc patients (Figure 3D). To further characterize this finding, we studied in a subset of seven patients and eight healthy subjects the frequency of CD25^high^CD27^high^CD86^high^CD1d^high^ B cells, since this population has been described to express high levels of IL-10 (and also TGF-β) and to exhibit strong regulatory properties (20). In accordance with the decreased frequencies of IL-10^+^ B cells, SSc patients presented reduced percentages of CD25^high^CD27^high^CD86^high^CD1d^high^ B cells compared with healthy controls (Figure 3E).

### B cells from systemic sclerosis patients exhibit a shift in the balance of activating and inhibitory receptors

**Figure 4:**
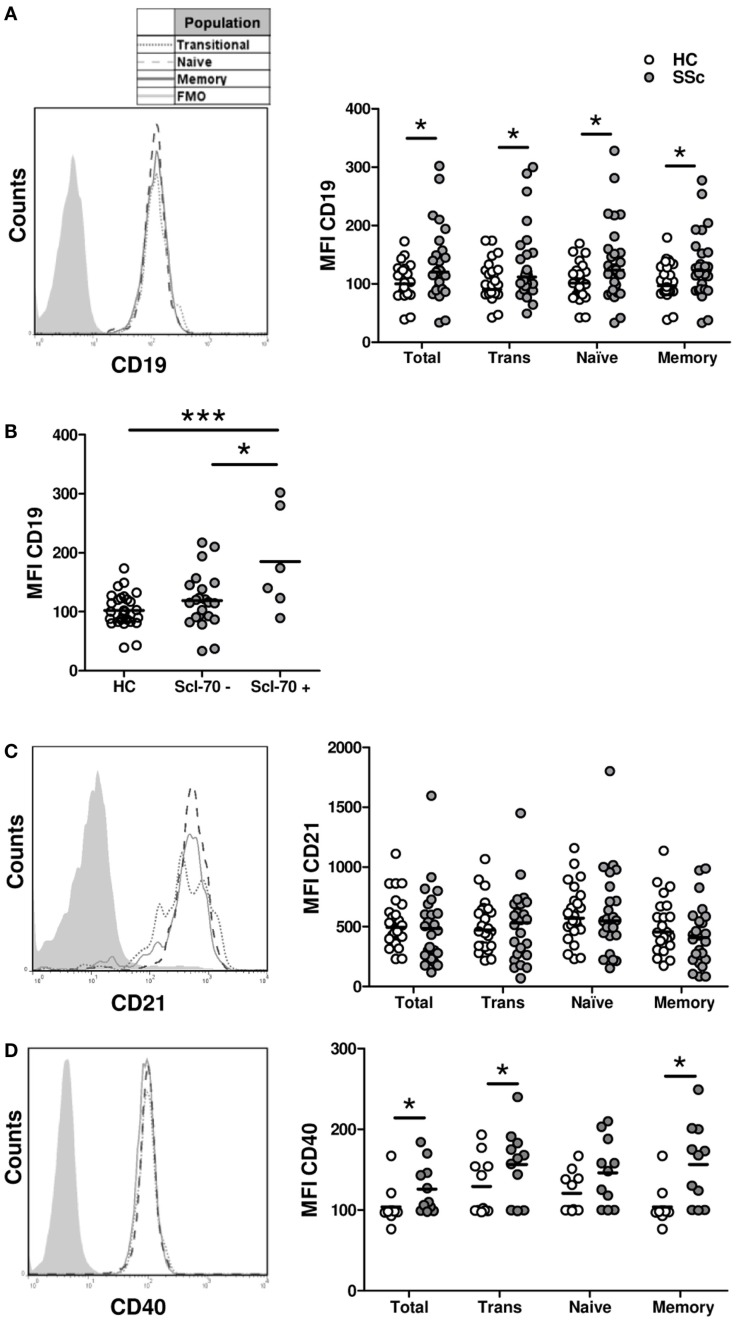
**Expression of the activation molecules CD19 (A,B), CD21 (C), and CD40 (D) in B cells from systemic sclerosis patients (*n* **=** 29, 24, and 19, respectively) and healthy subjects (*n* **=** 28, 24, and 19, respectively)**. **(A,C,D)** Left: representative histograms of the expression of each molecule on transitional (dotted line), naive (dashed line), or memory B cells (solid line). The shaded curve represents the fluorescence minus one (FMO) control staining. Right: graphs summarizing the expression of each molecule on total CD19^+^ B cells, transitional B cells (Trans), naive B cells, and memory B cells in healthy controls (HC, white circles) and systemic sclerosis patients (SSc, gray circles). **(B)** Expression of CD19 in total B cells from HC and SSc patients classified according to the presence of anti-Scl-70 antibodies. *P < 0.05, **P < 0.01, ***P < 0.01. Mann–Whitney *U* test for graphs in **(A,C)**, Wilcoxon signed-rank test for graphs in **(D)**, and unpaired Student’s *t*-test for graph in **(B)**. MFI, mean fluorescence intensity.

The activated phenotype displayed by B cells from SSc patients could be caused by an overexpression of molecules involved in B-cell activation. To test this possibility, the expression of three molecules that participate in B-cell activation was measured by flow cytometry: CD19, CD21, and CD40. In accordance with previous reports (21), B cells from SSc patients displayed a high expression of CD19 not only in naive and memory subpopulations but also in the transitional subset (Figure 4A). The expression of CD19 on total B cells and on each subpopulation was even higher in a subset of SSc patients with anti-Scl-70 antibodies, which is mainly associated with dcSSc (Figure 4B). In contrast, no differences were observed when comparing CD21 expression on different B-cell subpopulations from SSc patients and healthy controls (Figure 4C). Regarding CD40, increased expression levels were observed in total B cells as well as in all B-cell subpopulations from SSc patients (Figure 4D).

**Figure 5:**
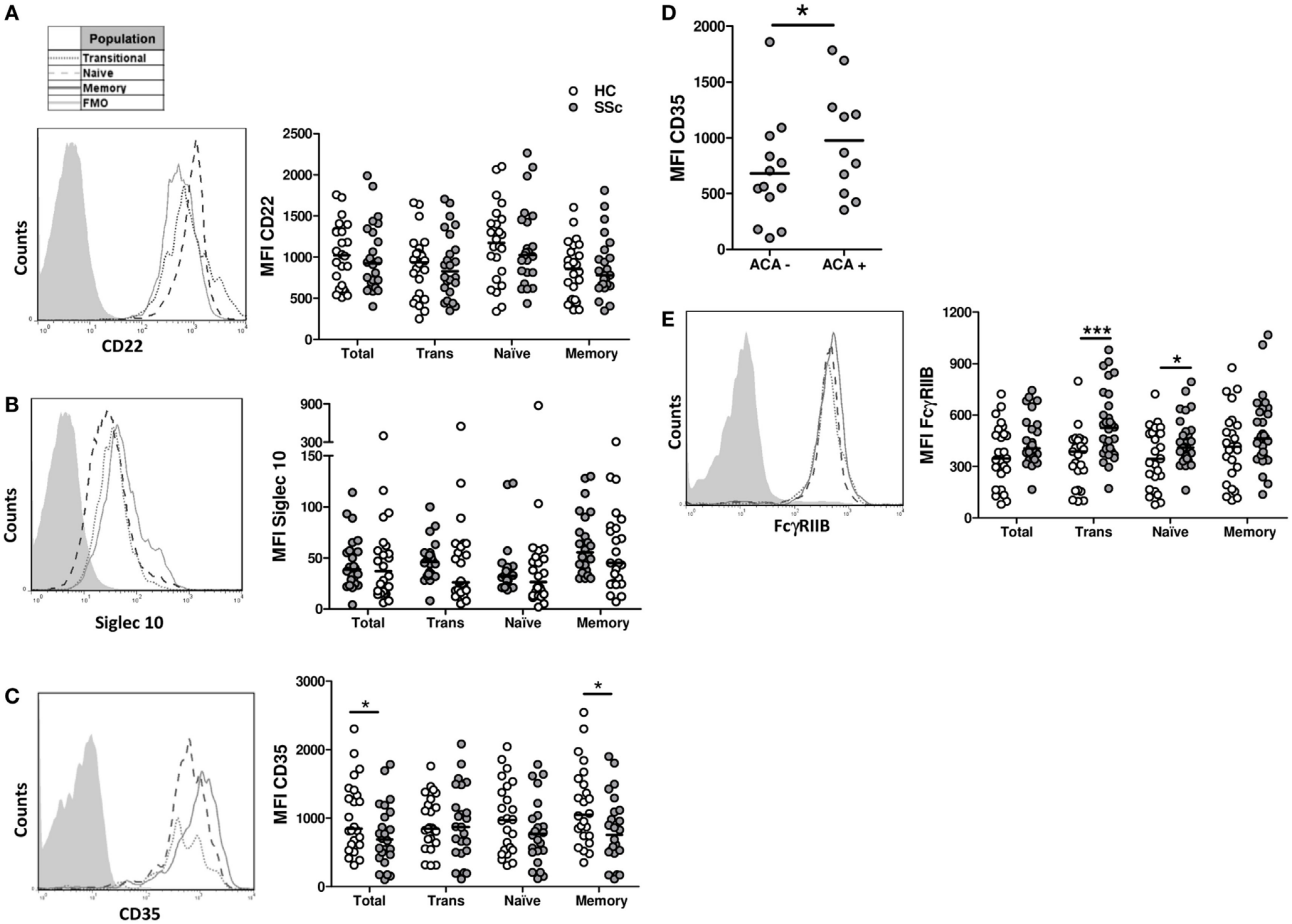
**Expression of the inhibition receptors CD22 (A), Siglec 10 (B), CD35 (C,D), and Fc**γ**RIIB (E) in B cells from systemic sclerosis patients (*n* **=** 24, 24, 24, and 30, respectively) and healthy subjects (*n* **=** 24, 24, 24, and 25, respectively)**. **(A–C,E)** Left: representative histograms of the expression of each molecule on transitional (dotted line), naive (dashed line), or memory B cells (solid line). The shaded curve represents the fluorescence minus one (FMO) control staining. Right: graphs summarizing the expression of each molecule on total CD19^+^ B cells, transitional B cells (Trans), naive B cells, and memory B cells in healthy controls (HC, white circles) and systemic sclerosis patients (SSc, gray circles). **(D)** Expression of CD35 in SSc patients according to the presence of anticentromere antibodies (ACA). *P < 0.05, ***P < 0.001. Paired Student’s *t*-test for graphs in **(A,C)**, Wilcoxon signed-rank test for graphs in **(B)**, and unpaired Student’s *t*-test for graphs in **(D,E)**. MFI, mean fluorescence intensity.

**Figure 6:**
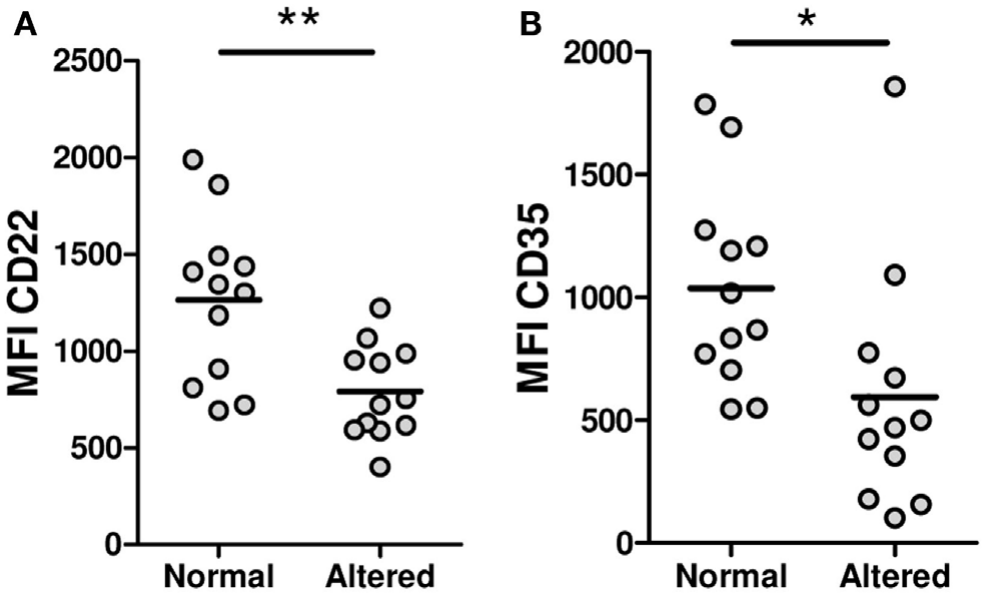
**Expression levels of CD22 (A) and CD35 (B) on total B cells from systemic sclerosis patients with a peripheral vascular Modified Medsger scale value **=** 0 (normal) or **≥**1 (altered)**. *P < 0.05, **P < 0.01, unpaired Student’s *t*-test. MFI, mean fluorescence intensity.

To find out if a defective expression of inhibitory receptors could be associated with the B-cell hyperactivity observed in SSc patients, the surface expression of CD22, Siglec 10, CD35, and FcγRIIB was examined on B cells from SSc patients and healthy subjects. Neither CD22 nor Siglec 10 showed altered expression levels in any of the B-cell subpopulations from SSc patients that were studied (Figures 5A,B). In contrast, SSc patients exhibited lower levels of CD35 expression in CD19^+^ B cells, in particular in the memory compartment (Figure 5C). Moreover, the subset of patients with anticentromere antibodies, which associate with lcSSc, showed an increased expression of CD35 in total B cells and in all subpopulations (Figure 5D). Unexpectedly, FcγRIIB expression was found to be significantly increased on naive and transitional B-cell subsets, but not on memory B cells, from SSc patients (Figure 5E). Of interest, patients exhibiting peripheral vascular alterations, as assessed by the Modified Medsger scale, showed a low expression of CD22 and CD35 in total B cells and in all B-cell subpopulations (Figure 6), suggesting a critical role of inhibitory molecules on B cells in the vascular component of this disease.

## Discussion

In the present study, we found important alterations in the frequency of different B-cell subpopulations and in the balance between activating and inhibitory molecules expressed by these B-cell subsets in SSc patients. SSc patients exhibit a bias in the distribution of B-cell subpopulations toward an increase in the relative frequency of naive cells and a reduction of the memory compartment. These results, as well as the increase in the percentage of CD19^+^ B cells within PBMC, are in agreement with a previous study in Japanese patients (21). However, this study describes for the first time an increase in the proportion of the transitional B-cell subpopulation in SSc patients. Similar findings have been shown for other autoimmune diseases, such as SLE and primary Sjögren’s syndrome (22).

Although it has been suggested that an expansion of immature forms of B cells in these conditions could be caused by failures in early tolerance checkpoints (23), some of these autoreactive transitional B cells could correspond to IL-10-secreting regulatory B cells. Furthermore, we found a reduced percentage of IL-10^+^ B cells in the peripheral blood of SSc patients, not only in the transitional subpopulation but also in memory and naive B cells. Moreover, SSc patients also exhibited reduced percentages of CD25^high^CD27^high^CD86^high^CD1d^high^ B cells, a B-cell subpopulation able to suppress CD4^+^ T-cell proliferation (20). Alterations in regulatory B-cell functions have been observed in other autoimmune diseases (16, 17, 24), and SSc may not be an exception; however, functional analysis, such as T-cell responses-inhibition assays, should be performed before drawing such conclusion.

B cells from SSc patients have been reported to present an activated phenotype, which leads to an overproduction of Ig, including autoantibodies, and an active production of cytokines, such as profibrotic IL-6 (25). In the present study, we demonstrated that naive and transitional B cells from SSc patients exhibit an activated phenotype, revealed by an increased expression of CD86 compared with healthy controls. In contrast with previously reported results, this difference was not detected in memory B cells (21). Resting naive B cells have been described to cause an incomplete activation of T cells, in part, due to the lack of expression of CD86, which can be restored after stimulation (26, 27). Therefore, an upregulation of CD86 on activated naive B cells could contribute to SSc pathogenesis by activating autoreactive T cells, which in turn stimulate the secretion of profibrotic cytokines by fibroblasts (28). In contrast, regulatory B cells expressing markers of transitional B cells are active promoters of tolerance, as they are able to induce regulatory T cells, a function that is largely dependent on CD86 (24). The impact of the increased CD86 expression found in transitional B cells from SSc patients requires further investigation.

The activated phenotype of B cells in SSc has been attributed to an increased expression of the activating molecules CD19 and CD21 (29). In addition, a polymorphism in the CD22 coding gene that is associated with a decreased expression of this receptor on B cells has been reported to be more frequent in a subset of Japanese lcSSc patients (30). Similarly, in the *tight skin* TSK/+ murine model of SSc, hyperresponsive B cells depend on an exacerbated activity of CD19 and an impaired counterregulation by CD22 (31, 32). In the results presented herein, an increased expression of CD19 and CD40, but not of CD21, was found in SSc B cells. The differences observed between this study and previous ones, regarding the expression of B-cell surface molecules such as CD40, CD21, and CD86 or the secretion of IL-10 and IL-6 by SSc B cells, could be attributed to different experimental settings or to the composition of the study group, in terms of the proportion of patients presenting lcSSc or dcSSc, as well as the undergoing therapy (21, 29). Indeed, decreased expression of CD40 and augmented levels of CD22 were found in patients receiving steroids (data not shown).

On the other hand, although no differences were found in the expression of CD22 or Siglec 10 on B cells from SSc patients or healthy subjects, a reduced expression of CD35 was detected in memory B cells from SSc patients. A reduced expression of this inhibitory complement receptor has been previously reported for other autoimmune diseases, such as SLE and RA, but never before for SSc (33–35). On human B cells, CD35 inhibits the BCR- and CD40-induced increase in cytoplasmic Ca^2+^ levels, proliferation, and antibody secretion (11, 33). This suggests a role of CD35 as a late checkpoint in preventing the maturation and differentiation of autoreactive B cells, a function that could be altered in autoimmune diseases such as SSc (6). In this sense, it is remarkable to note that in this group of patients, expression levels of molecules involved in the regulation of B-cell activity are associated with different subsets of patients. For instance, patients carrying anti-Scl-70 antibodies, which are characteristic of dcSSc, exhibited high expression levels of CD19 while patients carrying anticentromere antibodies, which are related to lcSSc, showed increased expression levels of CD35. The immunologic basis of these findings remains obscure for us.

Intriguingly, naive and transitional B cells from SSc patients showed high expression levels of the inhibitory receptor FcγRIIB. Our group and others have described that in autoimmune diseases, such as RA and SLE, B cells exhibit a reduced expression of this receptor in the memory subpopulation, associated to high levels of autoantibodies (34–38). Moreover, the absence of FcγRIIB specifically on B cells predisposes for the development of lupus and arthritis in animal models, highlighting its role as a crucial molecule for the control of autoimmune humoral responses (39). Although circulating human transitional B cells have been initially described to express moderate levels of FcγRIIB, the function of this receptor in transitional B cells remains unknown (22). A more recent publication reports a subpopulation of mouse IL-10-secreting B cells that expresses high levels of FcγRIIB, which endows these cells with the ability for an efficient endocytosis of immune complexes and inhibition of CD4^+^ T-cell responses. The authors postulate that by capturing immune complexes, these FcγRIIB^high^ B cells may attenuate the activation of immune responses (40). It remains unclear how the increased expression of FcγRIIB on the transitional subpopulation could be involved in the alterations observed in SSc B cells. One possibility is that it may be a compensatory mechanism triggered after the development of an exacerbated humoral response. Another explanation is that FcγRIIB could inhibit IL-10 secretion by transitional B cells after binding autoantibody-containing immune complexes, thus precluding its regulatory functions.

Altogether, this study demonstrates alterations in the frequencies and activation status of different B-cell subpopulations from SSc patients, including for the first time an analysis of the transitional B-cell subpopulation. We are currently carrying out *in vitro* experiments in order to uncover the mechanisms through which these abnormalities affect the function of B cells in this disease as well as key signaling molecules involved.

## Conflict of Interest Statement

The authors declare that the research was conducted in the absence of any commercial or financial relationships that could be construed as a potential conflict of interest.

## Funding

This work was supported by FONDECYT-Chile (grant nos. 1121100 and 11121497), REDES 140041 and the Millennium Institute on Immunology and Immunotherapy P09-016-F.
